# Nutrition programmes for individuals living with disadvantage in supported residential settings: a scoping review

**DOI:** 10.1017/S1368980022000969

**Published:** 2022-09

**Authors:** Verena T Vaiciurgis, Karen E Charlton, Annabel K Clancy, Eleanor J Beck

**Affiliations:** 1 School of Medical, Indigenous and Health Sciences, University of Wollongong, Wollongong, NSW 2522, Australia; 2 Illawarra Health & Medical Research Institute, Wollongong, NSW 2522, Australia

**Keywords:** Nutrition education, Disadvantage, Intervention, Homeless shelter

## Abstract

**Objective:**

Health inequities such as chronic disease are significantly higher among individuals living with disadvantage compared with the general population and many are reported to be attributable to preventable dietary risk factors. This study provides an overview of the current nutrition interventions for individuals living with extreme disadvantage, in supported residential settings, to develop insights into the development and implementation of policies and practices to promote long-term nutritional health and well-being.

**Design:**

A scoping review searched Scopus, ProQuest, CINAHL Plus, MEDLINE, and Web of Science databases using the terms ‘resident’, ‘nutrition’, ‘disadvantage’, ‘intervention’ and their synonyms, with particular emphasis on interventions in residential settings.

**Setting:**

Residential services providing nutrition provision and support.

**Participants:**

People experiencing extreme disadvantage.

**Results:**

From 5262 articles, seven were included in final synthesis. Most interventions focused on building food literacy knowledge and skills. Study designs and outcome measures varied; however, all reported descriptive improvements in behaviour and motivation. In addition to food literacy, it was suggested that interventions need to address behaviour and motivations, programme sustainability, long-term social, physical and economic barriers and provide support for participants during transition into independent living. Socio-economic issues remain key barriers to long-term health and well-being.

**Conclusions:**

In addition to food literacy education, future research and interventions should consider utilising an academic-community partnership, addressing nutrition-related mental health challenges, motivation and behaviour change and a phased approach to improve support for individuals transitioning into independent living.

It is well established that poor diet quality is a key modifiable risk factor for non-communicable diseases^(1)^. Despite this, globally 11 million deaths and 255 million disability-adjusted life-years are reported to be attributable to preventable dietary risk factors^(1)^. It is also well established that health inequities are significantly higher among individuals living with disadvantage (vulnerable individuals) compared with the general population, including chronic disease, disability and early mortality^(2,3)^. These inequities are greater in individuals experiencing extreme disadvantage and at-risk of, or experiencing, primary, secondary or tertiary homelessness^(4)^. This population includes highly marginalised groups such as people living with severe mental and behavioural health disorders, racial/ethnic minorities, victims of family and domestic violence, people with a history of substance abuse disorders, Indigenous peoples and individuals released from incarceration^(3,5)^. These individuals are also less likely to access and afford standard health care, which is often not tailored to their needs, suggesting that new modalities must be considered.

A recent systematic review and meta-analysis found that individuals from high-income countries experiencing homelessness, substance abuse disorders and incarceration have a mortality rate around eight times higher for men, and twelve times higher for women, compared with the general population^(6)^. These health-related disparities are likely the result of a complex range of uncertain social, physical, cultural and economic factors^(5)^. Dietary intake is influenced by all of these factors leading to unfavourable differences in dietary intake, dietary behaviours and overall dietary patterns. Consequently, adverse health outcomes are more likely including higher burden of disease incidence, morbidity, mortality and reduced quality of life^(7)^. Specifically, male homeless clients utilising residential services were likely to have a history of chronic alcohol abuse (62 %), and/or other substance abuse (66 %) as well as mental health disorders (64 %)^(8)^. Other common health conditions included features of metabolic syndrome (44 %); CVD (38 %) and hepatitis C (29 %)^(8)^. Examples of residential services for the purposes of this paper include the provision of emergency or short-term accommodation including crisis shelters, temporary housing, crisis accommodation, emergency housing, night shelters, refuges, emergency accommodation, hostels for the homeless and transitional housing^(9)^.

Evidence demonstrates that nutrition interventions are highly effective in the treatment of mental health disorders, cardiometabolic disorders and alcohol and/or substance abuse^(10–16)^. Thus to address the multifactorial health inequities, nutrition should be considered as an integral component for improving the health status and quality of life in populations experiencing extreme disadvantage^(10–16)^. Despite this, a limited body of research exists around longer-term effective strategies to address nutritional health and well-being, and little has been done in collaboration with individuals within a residential setting^(17,18)^.

For populations experiencing disadvantage accessing residential support services, who are dependent on food provided in residential care and likely to suffer food insecurity when they leave the facility, it is important to review and better understand the factors influencing dietary intake to inform potential strategies to address nutrition-related health concerns. While in residential care, food is at least transiently more secure. Adequate nutrition and dietetic intervention may provide integral support for improving the health status, well-being and quality of life in this population, and ideally equip individuals with skills to maintain this after they leave the supported setting. Thus, residential settings provide a unique opportunity to better understand the factors driving nutritional health in disadvantaged individuals to identify and inform potential strategies to address diet-related health inequities in a supported environment. Therefore, the aim of this review was to provide an overview of the current evidence regarding nutrition interventions conducted within residential settings.

## Methods

### Protocol and registration

The study protocol was preregistered with the Open Science Framework (10.17605/OSF.IO/ZSD6F), and findings were reported according to the Preferred Reporting Items for Systematic Reviews and Meta-analyses extension for scoping review (PRISMA-ScR) guidelines^(19)^. We searched for relevant research which had implemented nutrition interventions in residential care for people living with disadvantage.

### Information sources and search strategy

A scoping review was conducted using the five stage framework developed by Arksey and O’Malley, including recommendations by Levac *et al.,* Pham *et al.* and Peters *et al.* to enrich the methodology^(20–23)^. One author (VV) conducted the initial search up to April 30, 2021. Electronic databases searched included Scopus, ProQuest, CINAHL, MEDLINE and Web of Science with no limits on study design, date or language. Search terms were informed by the research question and included ‘residen*’, ‘housing’, ‘food’, ‘nutrit*’, ‘diet’, ‘vulnerable’, ‘disadvantage’, ‘socio-economic’, ‘homeless’, ‘low income’, ‘marginal*’, ‘education’, ‘program’, ‘literacy’, ‘food *security’ and ‘food assistance’. To ensure the search strategy was comprehensive in identifying all potentially relevant published and unpublished primary studies, the original search was supplemented by scanning the reference lists of relevant reviews, hand-searching of key journals and an online search of Google search operators, Australian charitable and government organisation websites^(20)^.

### Eligibility criteria

To be included in this review, papers needed to: (i) be a primary study and include; (ii) exposure to a nutrition-related intervention in a residential setting; (iii) reported nutrition-related outcome or results and (iv) participants identified as disadvantaged group. Studies were excluded for residential aged care and disability settings and interventions focused solely on children aged 12 years and below. For studies of interventions that addressed multiple health risk behaviours (i.e. an intervention targeting both diet and physical activity), only information pertaining to the nutrition-related outcomes was included.

### Study selection and data extraction

All relevant citations were collated into EndNote version X9, and exported to Covidence systematic review software (Veritas Health Innovation, Melbourne, Australia, 2020) where duplicates were automatically removed. To determine eligibility, two researchers (VV and EB) applied the inclusion and exclusion criteria to all titles and abstracts. Discrepancies were resolved by two researchers (VV and AC) and copies of the full-text articles were obtained for the remaining studies. Two researchers (VV and EB) strictly applied inclusion and exclusion criteria to determine final included studies. The extracted data included specific details of the participants, setting, study design, methods, intervention details (length, duration, concepts) and key findings synthesised according to the review question. The data extraction template was initially developed and charted by one researcher (VV) and continuously reviewed and updated by consensus among all researchers. Using qualitative synthesis and assessment, interventions were deemed to be successful if they reported positive changes in one or more of the outcomes of interest. Interventions that were classified as being successful were scrutinised for key characteristics of the intervention that contributed to their success, and this information is presented in order to inform recommendations for future programmes. Conversely, any noted barriers that contributed to a lack of change in outcomes were also identified. This data extraction was performed independently by one researcher (VV) and then discussed among the research team to reach consensus.

## Results

### Search strategy results

**Fig. 1:**
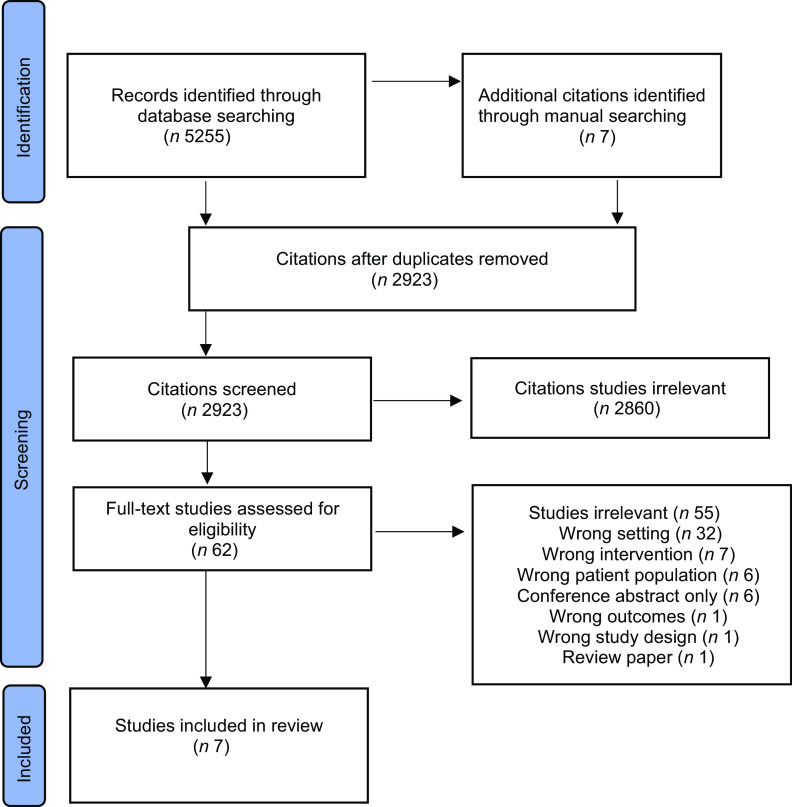
Preferred reporting items for systematic reviews and meta-analyses extension for scoping review (PRISMA-ScR) of included articles relating to nutrition interventions in residential care for individuals living with disadvantage

In total 5255 titles were identified and an additional seven studies were found through manual searching of references lists and key journals by one researcher (VV). After removal of duplicates, 2923 papers were screened on title and abstract of which 62 were selected for full-text review. Seven papers were finally included using the strict criteria, with the most common reason for exclusion being that the intervention was not conducted in a residential setting (Fig. 1).

### Description of included studies

The seven included papers reported on six nutrition interventions. The duplicate study had one paper reporting the initial intervention, and a second paper as a description of the outcomes. Both papers were included to give sufficient details on both interventions and outcomes. Papers were published between 2006 and 2020, with four studies conducted after 2017. Three of the interventions were conducted in Australia and the other three in the USA.

The majority of interventions were targeted towards people experiencing or at-risk of homelessness (*n* 4), one for low socio-economic adults and one for youth (>12 years) in-out-of-home care and their carers. All interventions were available in a residential setting; one community-based youth housing, two transitional homeless shelters, one emergency housing programme and two were established external programmes available to be delivered across multiple sites in a variety of settings, including residential care settings such as rehabilitation and housing support services. Intervention programmes ranged from four to twelve sessions, with a duration range of 1 to 3 h/session. Three of the programmes involved a dietitian or nutritionist, two utilised existing staff (either a caseworker or nurse practitioner) and one was designed and facilitated by occupational therapists (Table 1).

**Table 1:** Summary of study and intervention characteristics for included studies in scoping review on nutrition interventions in residential care for individuals living with disadvantage (*n* 7)

Reference; year, country, name of programme	Type of study	Aim of intervention	Setting/provider (if specified)	Target group/sample	Duration	Main outcomes
1. Meiklejohn *et al*. 2017, Australia, FoodMate Programme^(23)^	Qualitative pilot case study	Explore experiences/impacts of a nutrition intervention on staff and graduates	Community-based youth housing agency Delivered by agency caseworkers (trained by dietitians)	At-risk’ youth experiencing homelessness (*n* 10 programme graduates, *n* 5 youth service staff)	8 × 3 h weekly sessions	Platform for social engagement Decreased reliance on emergency food relief Developed food-related knowledge and skills A step towards food security Positive, sustained impacts of dietary behaviours up to 2 years Personal intrinsic motivating factors enabled sustained behaviour change
2. Yousey *et al.* 2007, USA, Early Childhood Enhanced Health Programme^(24)^	Descriptive project: Implementing and evaluating an educational programme	Improve the nutritional status of homeless children aged 18 months to 6 years	Homeless shelter for women and children Registered dietitian (staff), clinic nurse OR nurse practitioner (mothers)	Mothers (*n* 56) Cafeteria staff (*n* 3)	Mothers: 4 × 1 h nutrition classes over 9 months Staff: 3× nutrition classes over 4 months	Mothers significant improved nutritional knowledge: pretests 4·60, posttests 6·30 (*P* < 0·001), No change in portions sizes, nutritional quality of foods served observed post-staff education Reported staff constraints: budget, food donations (hinder nutrition) 50 % (*n* 28) mothers completed all four modules
3. Kendzor *et al*., 2017, US^(26)^	Randomised control trial	Evaluate the feasibility and effectiveness of a diet and physical intervention activity intervention for homeless adults	Transitional shelter	Homeless adults shelter residents (*n* 32) Intervention: *n* 17 Control: *n* 15	4-week diet and physical activity programme Intervention Group 4× Tailored Educational Newsletters, Daily fruit and vegetable snacks offered two daily 5/7 Control Group Assessment only	No significant difference: fruit and vegetable consumption, weight, waist circumference (all *P* ≥ 0·73) between groups 93·8 % (*n* 30) food insecure, (18·8 % low food security and 75·0 % very low food security) Intervention group (*n* 17) attended an average of 8·56 snacks (sd = 7·04, range 0–22), which was 24·2 % (sd = 21·23 %) of all possible snacks (highest during first week 29·0 % and lowest 14·3 % during the final week 33·3 % positive change in motivation to increase fruit/vegetable intake (did not differ by group)
4. Helfrich *et al*., 2007, US^(25)^	Longitudinal design: variable exposure to a novel life skills intervention	Present outcomes of intervention to maintain residential stability and prevent homelessness for adults with mental illness, using empowerment and social learning theories to evaluate intervention Determine if adults with mental disorders at risk for homelessness could: (1) learn life skills in food and money management; and (2) retain their knowledge and skills 3 to 6 months after completing the programme	Emergency Housing Programme (*n* 28) Single-room occupancy housing (*n* 23) Occupational therapist	Homeless adults living with mental illness (*n* 51)	12 modules over 6 weeks, 6 × 60 min group and six × individual sessions	Improved skills in food and money management, no significant difference at 3 or 6 months No significant difference in outcomes between sites 65 % participants exhibited two to six conditions (cardiac, orthopedic, pulmonary or endocronogolical disorder) Age negative correlation to performance scores food (*r* = 0·0433, *P* = 0·034) and money management (*r* = 0·413, *P* = 0·032) post-intervention Educational level positively correlated with food (*r* = 0·345, *P* = 0·099) and money management (*r* = 0·319, *P* = 0·105) post-intervention
5. Helfrich *et al*., 2006, US^(29)^	Group pretest–posttest design	Present three exploratory studies of life skills interventions (employment, money management or food/nutrition)	Four shelters and supportive housing programmes Occupational therapy students (third year or masters programme	Homeless individuals living in supported accommodation (Food and Nutrition Intervention – Adults living with Mental Illness) Total all three interventions *n* 73	Eight sessions over 4 weeks 60-min group and a 60-min individual session each week	32/73 participants completed pre- and posttests including six youths, 13 women who experienced domestic violence and 13 adults with mental illness Descriptive increase in mastery scores over time, no statistical significance in nutrition intervention (adults with mental illness)
6. Cox *et al.*, 2017, Australia HEAL Study^(28)^	Randomised control trial	To measure the efficacy of the programme to help young people make positive choices—eating and physical activity behaviours, and resources provided to professional carers to model, encourage and support change.	Fourty-eight residential care units	Young people who live in residential out-of-home care (OOHC) and their carers 77 young people, 177 carers Intervention *n* 25 Control *n* 23	12-month programme (includes 6 months maintenance), 8 fortnightly sessions	118 carers and 51 young people lost to follow-up No significant differences between participants and lost to follow-up No evidence for efficacy of the intervention for either young people or carers Significant main effects; confidence to change (diet) (*β* = -0·77, *P* = 0·04), readiness (diet) (*β* = -2·09, *P* = 0·01), and unhealthy foods (*β* = 2·73, *P* = 0·03) Positive shift in behaviours in intervention group: decreased sugary drink consumption and BMI *Z*-scores, healthy food consumption (not statistically significant) No measurable effect on dietary, physical activity or weight outcomes for young people and their carers
7. West *et al*., 2020, Australia, NEST Programme^(27)^	Descriptive evaluation study, mixed-methods approach	Inquiry into value of NEST (Nutrition Education and Skills Training) programme in promoting food security and food literacy Identify barriers/enablers in sustaining food security and utilizing food literacy skills beyond the programme Improve nutrition, food literacy and cooking skills of low-socio-economic Australian adults.	Varied—programme travels to multiple settings e.g. rehabilitation, health services, food pantries, community centres and housing support services Facilitators are university-qualified nutritionists and dietitians	Low-socio-economic Australian adults	6 weeks, 15 h	Mean food security score significant decrease (28 %, *P* = 0·030), Statistically significant improvements: cooking confidence (*P* = 0·001)*, food preparation behaviours (*P* = 0·006)†, nutrition knowledge (*P* = 0·033), daily vegetable intake (*P* = 0·043), sugar-sweetened beverage consumption (*P* = 0·017), salty snack foods (*P* = 0·011) Interview Results (*n* 17): Demographics – male (64·7 %), (70·6 %), living in social housing or rehabilitation centres (58·8 %), household income of <$AUD 575/week (58·8 %), age range 24–80 years, mean age of 48·8 (±16·4) years Improved self-reported ability to stretch food budgets and for food utilisation Enablers of Food Security: (1) receiving and providing support to family or friends; and (2) provision of charitable food. Barriers to Food Security: (1) lack of economic access to food; (2) pre-existing health issues and (3) provision of charitable food

* All individual measures improved, except confidence in ability to buy healthy food on a budget.

† Most food behaviour scores were significant, except reading the ingredient list, looking at price per kilo when shopping, changing recipes to make them healthier and adding salt to food when cooking.

All interventions (*n* 6) included components of healthy eating, and most (*n* 5) included and addressed personal behaviours, motivation and readiness to change (Table 2)^(24–29)^. Most (*n* 4) included sessions topics related to fruits and vegetables, budgeting, practical cooking lessons and physical activity^(24–29)^ while three addressed food storage, food safety, meal planning and shopping strategies or tours^(24–26,28)^. Two of the interventions included food label reading, well-being, food swaps and recipe modification and offered a flexible structure where participants had the option of completing sessions individually, as part of a group, or a combination as guided by the participant^(24,25,28)^. Of the seventeen identified intervention session topics reported, one intervention included 15/17 and one 13/17 components^(24,28)^, while the remaining interventions included between four and nine topics. One intervention^(28)^ was reported to be underpinned by social cognitive theory with a focus on building self-efficacy and one based on empowerment theory and social learning theory^(26)^. The remaining studies did not specify a framework.

**Table 2:** Summary intervention components and topics included in the scoping review of studies on nutrition interventions in residential care for individuals living with disadvantage

Ref #	Healthy eating	Fruits and veg	Practical cooking lessons	Budgets	Food storage	Food safety	Physical activity	Meal planning	Label reading	Shopping strategy	Well-being	Food waste	Long-term barriers	Food swaps/recipes	Flexible format	Self-sustaining	Behaviour/motivation	Total *n* 17	%
23	✔	✔	✔	✔	✔	✔	✔		✔	✔				✔	✔	✔	✔	13	76·5
24	✔	✔		✔				✔							✔	✔		6	35·3
26	✔	✔					✔										✔	4	23·5
25	✔	✔	✔	✔	✔	✔		✔		✔							✔	9	52·9
28	✔		✔				✔				✔						✔	5	29·4
27	✔	✔	✔	✔	✔	✔	✔	✔	✔	✔	✔	✔	✔	✔			✔	15	88·2
Total *n* 6	6	5	4	4	3	3	4	3	2	3	2	1	1	2	2	2	5		

✔ = reported in study.

### Successful programme components

Although study designs were highly varied and all reported descriptive improvements, the study presenting the most substantial improvements in behaviour change, dietary intake and food literacy measures was provided by a charitable organisation, *OzHarvest’s NEST* programme^(28)^. This study used a mixed-methods approach to evaluate a 6-week public health nutrition programme aimed to address food insecurity for low-socioeconomic Australian adults, facilitated by university-qualified dietitians and nutritionists^(28)^. Each module included a lesson topic presentation and discussion, interactive practical activities, goal setting, practical cooking and sharing a meal. Weekly teachings were designed to be non-judgemental and ensure participants felt included and welcomed by focusing on healthy positive behaviour change and encouraging group discussion. Statistically significant outcomes included improvements in overall measures of food security (*P* = 0·03), cooking confidence (*P* = 0·001), health-promoting food behaviours (*P* = 0·006), nutrition knowledge (*P* = 0·033), daily vegetable intake (*P* = 0·043) and reduced sugar-sweetened beverage (*P* = 0·017) and salty snack food consumption (*P* = 0·011)^(28)^. Qualitative results identified that these improvements were attributable to enhanced food literacy and budgeting skills which lead to positive changes in food utilisation^(28)^. Authors described the mixed-methods design to be beneficial for exploring efficacious outcomes, however as the programme included food provision through cooking workshops, it was difficult to attribute the determining factors of success^(28)^. Authors further identified potential long-term issues for participants’ ability to afford/access ‘healthful’ foods beyond the programme, with most reporting they were still accessing highly varied (in both quality and quantity) food from charitable sources, which educational interventions alone cannot address^(28)^.

Similar to *NEST*, the *FoodMate* ^*TM*^ programme involved a non-profit food organisation *(SecondBite),* that was supported by universities and dietitians in design and facilitation and reported improved dietary behaviour changes, sustained up to 2 years^(24)^. This qualitative pilot study investigated the impacts of an eight-session nutrition education intervention addressing food insecurity for young people experiencing homelessness within existing case management services. Findings highlighted that the intervention provided ‘*a platform for social engagement’*, '*reduced reliance on emergency food relief’, ‘developed food related knowledge and beliefs’* and *‘a step toward food security’* for young people experiencing disadvantage. Specifically, participant and staff interviews identified that shared cooking and dining experiences provided opportunities for peer-to-peer support, friendship development and benefits from the ability to talk to people experiencing similar challenges^(24)^. Participants reported improvements in shopping strategies, takeaway food purchasing, food storage, cooking and eating habits, meal patterns, discretionary food consumption, budgeting skills and an increased motivation for behaviour change to prepare meals^(24)^. Additionally, a key difference in identifying participants that demonstrated sustained behaviour changes compared with those that did not was related to individuals’ level of pre-existing motivation and readiness to change. This is an important consideration when designing and delivering an intervention^(24)^. Embedding the intervention within existing case management services and provision of a flexible structure, namely one-on-one or group facilitation options, were also found to be key components to the programme’s success due to an ability to engage hard-to-reach, shy and tentative participants^(24)^. Similar to th*e NEST* programme, authors identified that participants reported being transiently food secure despite continuing to access food relief^(24)^.

Another intervention aiming to improve the nutritional status of homeless children, considered programme sustainability and was designed by a dietitian to be maintained without additional or new staff^(25)^. This programme consisted of four modules provided over a 9-month period that involved nutrition education for mothers, facilitated by clinic nurses and also shelter cafeteria staff, facilitated by the dietitian^(25)^. This programme was found to improve the nutrition knowledge of mothers, however had no impact on the nutritional quality of foods served by staff^(25)^. Budget was found to be the key driver explaining the lack of effect in staff-related outcomes. The timing of the programme coincided with a natural disaster and the shelter being filled beyond capacity, with no additional food budget allocated^(25)^. Authors noted that the intervention addressed knowledge, but did not consider additional factors that would enable participants to put their new knowledge into practice such as access to cooking facilities, meal preparations and the affordability of food^(25)^.

The two studies with the strongest study design were randomised controlled trials^(27,29)^. Both reported no significant differences on dietary or anthropometric outcomes; however found positive effects for behaviour and motivation to change^(27,29)^. The *HEAL* study^(29)^ aimed to measure the efficacy of eight fortnightly sessions provided over a 12-month programme (including 6 months of maintenance) in residential care units. Educational sessions were provided to: (1) young people focusing on positive choices for eating and physical activity behaviours; and (2) professional development for carers to support and encourage client change^(29)^. Authors reported challenges with recruitment, participation and retention due to the transient nature of the population and suggested this may be the reason for null effects. Authors further noted the particular challenges with recruiting and retaining participants and data collection and noted the importance of considering study design and flexible methods of data collection in this complex population group^(29)^. Similar issues with low retention rates (i.e. 34 %–50 %) were reported in two additional interventions in this review^(25,29,30)^.

The second randomised controlled trial was a 4-week diet and physical activity intervention designed to evaluate the feasibility and effectiveness of a programme for homeless adults living in a transitional shelter^(27)^. The intervention group received four tailored educational newsletters and were offered fruit and vegetable snacks twice daily on weekdays^(27)^. This study reported no significant outcomes for fruit and vegetable consumption or anthropometric measures and similarly, poor, diminishing attendance at snack time, however did report a 33 % change in reported motivation to increase fruit and vegetable intake^(27)^. Authors also noted barriers for residents around utilisation as they typically receive meals prepared by the shelter, and also that intervention benefits may diminish after leaving the shelter^(27)^. Of interest, they suggested a phased intervention approach to first address lifestyle risk factors within the residents, followed by a transitional phase preparing for independence, may have better supported participants in achieving and maintaining health long term^(27)^.

A number of studies noted improvements in motivation, food acquisition, nutrition knowledge, food preparation skills and budgeting^(25–27,29–31)^. As with other outcome interventions, where improvements were tracked over time, these improvements were difficult to sustain. For example, an exploratory study by Helfrich *et al*. provided life skills interventions to adults living with mental illness which included employment opportunities, money management or food/nutrition. While the study showed improvements in budgeting and food literacy initially, at 3 and 6 months, these improvements were not sustained^(26,30)^.

### Programme barriers

Most studies identified multiple barriers for participants, in particular, difficulties in sustaining behaviours and skills, motivation, food security and utilising learnt skills beyond programme completion^(24–26,28)^. Reasons identified were largely a lack of economic access to food and poor motivation related to mental health disorders and pre-existing health issues^(24–26,28)^. It was also identified that, despite self-reported food security, many individuals continue to depend on or access food assistance beyond the programmes^(24,25,28)^. Issues were also found with the validated tools measuring food security such as the Six-item USDA Short Form Food Security Survey Module which does not consider frequency of obtaining charitable food^(28)^. One study however did report this reliance on emergency food relief, to be potentially out of 'habit not need’ and due to a perceived inability to consume a well-balanced diet without emergency food relief^(24)^. This created anxiety in some participants, who despite their increased health literacy, were unable to utilise their new skills to provide nutritious meals for themselves and their children due to the types of charitable foods they were receiving and a lack of finances^(25)^. Staff at a youth shelter identified these limited opportunities and a lack of good role modelling makes long-term behaviour change particularly challenging for disadvantaged youth^(24)^. Despite these shortcomings, staff also viewed nutrition programmes as ‘planting the seed’ for gaining key knowledge and skills necessary to become food secure, but cautioned that this would likely take a long time as many were not yet independent, and may revert back to old behaviours^(24)^.

Overall, successful interventions generally involved a dietitian in their design and/or facilitation^(30)^, as well as tertiary education/university sector support to assist in programme design, facilitation and/or evaluation. Successful programmes considered the intrinsic motivation of participants and provided nutrition knowledge and skills through practical, interactive and experiential learning around the components of food literacy of planning and management, food selection, preparation and eating^(32)^. In designing interventions, the highly varied literacy and comprehension levels in this population group were an important consideration^(26,30)^. Multiple studies considered this through the provision of interactive and experiential learnings through games, practical sessions, photos, cookbooks and readability of language used in questionnaires or consideration of data collection using discrete methods such as observations and audits^(24,28–30)^.

## Discussion

This scoping review of nutrition programmes offered to disadvantaged individuals in residential settings found that favourable impacts were dependent on several personal and programme-related characteristics such as an underlying interest in nutrition and intrinsically motivated behaviours^(24,26–29)^. Interventions involving University and dietitian support, the provision of practical and experiential food literacy education and consideration of motivation and behaviour change presented the most successful results. These are also successful elements in nutrition interventions previously identified outside of residential setting^(33)^. It was also clear that, despite positive results in motivation, nutrition knowledge and food literacy measures, these interventions alone are not enough for achieving long-term health behaviours and outcomes. Improving individual knowledge and skills cannot address the complex social, environmental and economic factors limiting behaviour change, which are well-known drivers of food insecurity in low socio-economic groups^(34)^. These findings highlight a need for interventions to consider midstream and upstream social determinants of health^(35,36)^. Socio-ecological models or social-economic approaches in addition to local strategies and initiatives have been suggested in previous research to be useful in reducing the number of diet-related chronic disease in disadvantaged groups in the long term^(37–39)^. Therefore, to improve the dietary-related health status for these individuals, strategies need to also address the factors that impact behaviour, regardless of knowledge and skills, such as mental health difficulties and an inability to afford healthful foods.

Embedding interventions within existing services were found to lead to potential improved coordination of care^(40)^ and more sustained changes in food-related behaviours^(24)^, particularly for combined housing programmes involving case managers/caseworkers^(41)^ who are in an ideal position to provide ongoing client-centred support and reiterate key messages^(42)^. This also serves as an effective time for staff to initiate conversations to address, promote and fulfil requirements of their clients’ support plans. Similarly, conversations may establish post-programme pathways and coordination with other longer term support services. Importantly, these elements may extend beyond the reach of residential programmes into the community.

Academic–community partnerships are well established as a cost-effective approach for addressing nutrition challenges and public health disparities^(43–46)^. A large body of research involving academic–community partnerships has reported significant valuable benefits such as shared resources, building institutional capacity, additional funding, managing and enhancing new ideas, providing real-world learning opportunities for students’ skills and opportunities to extend and conduct new areas of research^(47,48)^. This approach allows for an exchange of ideas and expertise shared between universities and community members across all stages of the programme, from design, implementation and dissemination^(48)^. This also provides opportunities for train-the-trainer models, via student volunteers to enhance staff nutrition knowledge, self-efficacy and promote learning and skills beyond the lifetime of a single programme^(43–45)^.

Emerging research in nutrition and mental health, and the high prevalence of mental health disorders highlights the importance of maintaining motivation in this population group^(49–51)^. Opportunities exist to incorporate education to address specific nutrition challenges experienced by people living with mental illness such as reduced motivation, social exclusion and isolation and financial restraints^(52)^ to provide practical strategies to address them, particularly when living independently. The *NEST* programme was reported to be developing a mental health and well-being module^(28)^ while other programmes have noted a key objective of promoting social interaction^(53)^.

Similarly to previous research, another common challenge in this population for researchers was the recruitment and retention of participants^(54–56)^. The *HEAL* programme, provided residentially^(29)^, has also been applied in a non-residential setting^(57)^ with a much larger sample size. In this setting, improvements in all outcome variables (*P* < 0·001) for participants completing the programme were reported, including increases in daily serves of fruit and vegetables consumption, and reductions in body mass, BMI, waist circumference and blood pressure. This suggests that the residential setting study^(37)^ may have been underpowered. In an already underrepresented, often omitted population in public health and medical research^(58)^, and given that this group experience the highest burden of chronic disease, short-term support is not a longer term solution^(58,59)^. It is vital to accurately obtain detailed data and outcomes to accurately review interventions. Thus, research needs to address specific strategies to maximise participant retention and recruitments, in particular maintaining contact with people who are experiencing extreme disadvantage as they move beyond supported accommodation.

Transitions from residential services to independent living is a critical time^(60)^ for sustaining newly learned health behaviours, and many of this population group experience recurrent homelessness particularly those with a history of alcohol and substance disorders^(61)^. Evidence suggests a phased approach may be beneficial for maintaining longer term relationships and provides clients support to improve long-term health outcomes as well as increasing retention rates^(27,60)^. For example, the first phase, conducted in the residential setting, would involve addressing lifestyle risk factors through food literacy interventions. The second phase would support clients as they transition into independent living by focusing on practical strategies to achieve and maintain health behaviours including cooking and shopping on a budget, resource provision and follow-up appointments with a dietitian^(27)^. Involvement of multidisciplinary support, health services and social support services across both phases also have the potential to bridge the gaps in existing community services, which may not usually be available to these individuals.

There may also be other interventions conducted outside a residential setting that could be transferable to a residential setting. For example, a study showed benefits of a volunteer peer–teacher model, with improvements in nutrition knowledge around low-cost and low-fat meals and improved attitudes towards healthy, low-cost meal planning^(62)^. To address the potentially transient nature of the target population, another study targeted towards at-risk youth, designed their programme in collaboration with a health centre offering emergency food pantries via mobile and on-site clinics^(63)^.

### Strengths and limitations

Although some studies provided evidence for improvements in nutrition knowledge, skills and intrinsic motivation and behaviour, a limited number of nutrition interventions offered in residential settings exist for people experiencing disadvantage. This scoping review did not formally evaluate the quality of evidence. Of the cited studies, most did not have strong study designs and had small sample sizes, were often conducted in a single shelter with no control group and had limitations in study design. Participants were recruited through convenience sampling and data were typically observational, self-reported and subject to multiple biases including recall and participant bias. The transient nature of this population highlights difficulties with recruitment and retention, and all studies had issues with missing data. Thus, study findings to date warrant caution in their interpretation and application to practice. More research is required to better understand and make informed generalisable recommendations.

### Suggestions for future interventions

It is recommended that based on the studies reviewed, in addition to food literacy education, future research and interventions for people living with extreme disadvantage should consider nutrition-related mental health challenges^(52)^, motivation and behaviour change in participants which was found to be associated with improved outcomes. Given the majority of study designs were quasi experimental, consideration of more rigorous study methodologies such as a stepped-wedge cluster randomised trial in which residential settings are randomised would be beneficial for strengthening the current evidence base. Conducting a needs assessment is also recommended to identify the residents’ current skills, and their environment to ensure interventions match the clients’ priorities. Given that a critical time was highlighted as individual’s transitioned to independent living, it is possible that support during that time may be provided through the use of technology. It is also recommended that utilising an academic–community partnership, involving key stakeholders in design, implementation and evaluation such as onsite staff would be valuable for developing more relevant interventions, increasing participant and staff acceptability^(64)^, promoting ongoing support and socio-economic long-term programme longevity^(58)^.

## Conclusion

There is a lack of research on effective nutrition interventions undertaken in individuals living with extreme disadvantage in supported residential settings. Individuals in supported accommodation lack financial means to implement change and sustain positive nutrition behaviours, despite improved knowledge. Although some studies have provided evidence for improvements in nutrition knowledge, skills and intrinsic motivation and behaviours, interventions do not address long-term environmental and socio-economic factors. Individuals living with disadvantage require multi-modal and longer-term support strategies. In addition to food literacy education, future research and interventions should consider utilising an academic–community partnership, addressing nutrition-related mental health challenges, motivation and behaviour change and a phased approach to improve support for individuals transitioning into independent living.

## Supplementary material

For supplementary material accompanying this paper visit https://doi.org/10.1017/S1368980022000969. click here to view supplementary material
